# Comparison of the Staphylococcal Chromosome Cassette (SCC) *mec* in Methicillin-Resistant *Staphylococcus aureus* (MRSA) and Non-*aureus* Staphylococci (MRNAS) from Animals and Humans

**DOI:** 10.3390/antibiotics10030256

**Published:** 2021-03-04

**Authors:** Cyrille Ngassam Tchamba, Jean-Noël Duprez, Pierrick Lucas, Yannick Blanchard, Filip Boyen, Freddy Haesebrouck, Maria A. Argudín, Jacques Mainil, Damien Thiry

**Affiliations:** 1 Bacteriology, Department of Infectious and Parasitic Diseases, Faculty of Veterinary Medicine and Institute for Fundamental and Applied Research in Animals and Health (FARAH), University of Liège, Quartier Vallée 2, Avenue de Cureghem 6, 4000 Liège, Belgium; cngassam@student.uliege.be (C.N.T.); Jean-Noel.Duprez@uliege.be (J.-N.D.); damien.thiry@uliege.be (D.T.); 2 Viral Genetics and Bio-Security Unit, ANSES, Ploufragan-Plouzané Laboratory, Rue des Fusillés, 22440 Ploufragan, France; pierrick.lucas@anses.fr (P.L.); yannick.blanchard@anses.fr (Y.B.); 3 Department of Pathology, Bacteriology and Avian Diseases, Faculty of Veterinary Medicine, Ghent University, Salisburylaan 133, 9820 Merelbeke, Belgium; filip.boyen@ugent.be (F.B.); freddy.haesebrouck@ugent.be (F.H.); 4 Laboratory of Molecular Biology, Cliniques Universitaires Saint-Luc, Catholic University of Louvain, Avenue Hippocrate 10, 1200 Brussels, Belgium; maria.argudin@uclouvain.be

**Keywords:** *Staphylococcus aureus*, non-*aureus* staphylococci, methicillin resistance, *mecA* staphylococcal cassette chromosome, cattle, humans

## Abstract

Methicillin-resistant *Staphylococcus aureus* (MRSA) and non-*aureus* staphylococci (MRNAS) cause different infections in animals, including mastitis, in livestock and humans. This study aimed to identify and compare the staphylococcal chromosome cassette *mec* (SCC*mec*) types of MRSA or MRNAS isolated from several animal species and humans in different countries. Of 1462 *S. aureus* and non-*aureus* staphylococci, 68 grew on Chrom MRSA ID^®^ agar, were phenotypically resistant to cefoxitin and tested positive with the PCR for the *mecA* gene. These 60 MRSA and 8 MRNAS were isolated in Belgium mainly from cows (livestock-associated (LA) MRS) and humans (community-acquired (CA) MRS) and in Japan from dogs and cats. The SCC*mec* cassettes were identified by multiplex PCR in 52 MRSA and 7 MRNAS and by whole genome sequencing (WGS) in 8 additional MRSA. The SCC*mec* types IV and V were the most frequent in Belgian LA-MRS and CA-MRS, while the SCC*mec* type II was identified in four of the five Japanese MRSA. The remaining isolate was a bovine *S. haemolyticus* in which no SCC*mec* was identified. These results confirm the high prevalence of the SCC*mec* types IV and V in LA-MRS and CA-MRS in Belgium, emphasizing the possible public health hazard of the former, and the absence of SCC*mec* in some MRNAS.

## 1. Introduction

The history of methicillin-resistant *Staphylococcus aureus* (MRSA) began in 1961 following the development and marketing in 1959 of the methicillin antibiotic, a semisynthetic penicillin that is resistant to the activity of the β-lactamase (BlaZ) enzyme from *S. aureus* (SA) [1]. The mechanism of resistance of these first MRSA was not the production of any mutant Bla enzyme, but the synthesis of a β-lactam-resistant penicillin-binding protein (PBP2a), a transpeptidase involved in the bacterial cell wall formation, encoded by a newly acquired and chromosome-located *mec* gene [2,3]. The first MRSA were isolated from hospitalized human patients with nosocomial infections (hospital-acquired MRSA or HA-MRSA). During the 1990s, community-acquired MRSA strains (CA-MRSA) progressively emerged in humans with no history of hospitalization. HA-MRSA and CA-MRSA belong to different clonal lineages and virulotypes [4]. Though the first reported animal MRSA was isolated in 1972 from the milk of a cow with mastitis [5], they emerged in the early 2000s, not only in livestock but also in companion animals, horses and some wild animals and were generically named livestock-associated MRSA (LA-MRSA) [6].

Besides this first *mec* gene (renamed *mecA*), three other *mec* genes (*mecB, mecC* and *mecD*) have been identified as responsible for methicillin resistance in the *Staphylococcaceae* family [7,8,9]. The *mecB* and *mecD* genes were reported at first on the chromosome and/or on a plasmid of *Macrococcus caseolyticus* (previously named *Staphylococcus caseolyticus*). Recently, the *mecB* gene was also reported on a plasmid of one MRSA isolated from a human patient. Conversely, the *mecD* gene has not been reported in staphylococci so far [9,10]. Like for the *mecA* gene, the *mecC* gene was first reported in *S. aureus* isolates, though not from humans but from dairy cattle, and is also located on the chromosome [11]. These *mecA* and *mecC* genes are actually carried by a mobile genetic element named staphylococcal chromosome cassette (SCC) that is responsible for inter-*Staphylococcus* species transfer and, therefore, acquisition of methicillin resistance by non-*aureus* staphylococci (NAS), known as methicillin-resistant non-*aureus* staphylococci (MRNAS) [12,13]. Up to 14 different SCC*mec* have been described in MRSA, some of them being also present in MRNAS [11,14]. Nevertheless, the relative prevalence of these 14 SCC*mec* differs according to the host and country of origin of the MRSA or MRNAS [15,16,17]. Moreover, MRNAS can harbor additional SCC*mec* types that are not included in the official classification, and the *mecA* gene is not always located on an SCC*mec* in MRNAS [18,19]. 

The aims of this study were therefore: (i) to identify MRSA and MRNAS within collections from different hosts in European, African, Asian and North American countries using phenotypic and genetic assays; and (ii) to compare by multiplex PCR (mPCR) and whole genome sequencing (WGS) the SCC*mec* present in MRSA and MRNAS, focusing on bovine mastitis-associated staphylococci. 

## 2. Results

### 2.1. Screening and Identification

A total of 78 presumptive MRS(A) was obtained after screening 1462 SA and NAS isolates on Chrom MRSA ID^®^ agar: 61 SA and 8 NAS from Belgium, 3 SA from Niger, 1 SA from Canada and 5 SA from Japan. The 70 SA were confirmed by MALDI-TOF while the 8 NAS were identified to three species: *S. capitis* (one isolate), *S. epidermidis* (six isolates) and *S. haemolyticus* (one isolate). Of these 78 presumptive MRS(A), 60 SA and the 8 NAS were resistant to cefoxitin by the disk diffusion assay and the MIC (Minimal Inhibitory Concentration) test strips and *mecA* PCR-positive. These 68 *mecA*-positive staphylococci were isolated from cows, boar, horses, humans, fomites, dogs and cats in Belgium and Japan (Table 1). Conversely, seven SA were phenotypically sensitive to cefoxitin and *mecA*, *mecB* and *mecC* PCR-negative. The remaining three SA growing on Chrom MRSA ID^®^ were resistant to cefoxitin either by the disk diffusion assay or by the MIC test strips, but all three were PCR-negative for the three *mec* genes. 

### 2.2. SCC*mec* PCR

The multiplex PCR (mPCR) performed on the 68 *mecA* positive MRS(A) identified the SCC*mec* in 59 isolates: 9 isolates harbored the SCC*mec* type II, 2 isolates the type III, 34 isolates the type IV, 11 isolates the type V and 3 isolates the type VII (Table 2, Supplementary Table S1). The remaining nine MRS(A) were not typable (NT) by PCR including eight SA with a profile close but not identical to the SCC*mec* types II and IV, and one *S. haemolyticus* with a profile close to the SCC*mec* types VII and X. 

### 2.3. Whole Genomic Analysis

All genomic data related to this project, including raw reads, are available via the NCBI BioProject PRJNA607920. The genomic analysis of the eight SA and of the *S. haemolyticus* whose SCC*mec* could not be identified with the mPCR revealed that seven of the SA actually harbored one SCC*mec* type IV, and another one the SCC*mec* type II. Conversely, the *S. haemolyticus* harbored only a 100% sequence similarity *mecA* gene and a 99.68% sequence similarity IS*1272* gene, but no *ccr* complex nor any other *mec* complex elements (*mecR1/mec I*) (Table 2, Supplementary Tables S2 and S3). 

## 3. Discussion

The purpose of this study was to characterize and compare the SCC*mec* in MRSA and MRNAS from different hosts and countries isolated between 2005 and 2014 (see Materials and Methods Section 5.1). Unfortunately, only isolates from bovine mastitis and humans in Belgium—and from dogs and cats in Japan, to a lesser extent—tested positive phenotypically and genetically. Two methods were used for the identification of the SCC*mec* in the 68 MRSA and MRNAS studied. The mPCR results gave a perfect match with expected amplification results (Table 2) for 52 MRSA and 7 MRNAS, while the results were equivocal for 8 MRSA and 1 MRNAS. A final identification of the SCC*mec* in these eight MRSA was obtained by Whole Genome Sequencing (WGS) but not in the MRNAS (Table 2). Both the mPCR and the WGS indeed can present limits in the identification of the SCC*mec* not only in MRNAS, but even in MRSA. The limits of the mPCR are classically based on primer sequence mismatches, as consequence either of a point mutation or of the presence of a still untyped SCC*mec* [20]. WGS can also present some limits depending on the database used to identify the SCC*mec* [21].

According to the mPCR and to the WGS results, half of the SCC*mec* in the 26 MRSA from cattle (50%) belong to the type IV and the other ones to types II, V and VII (Table 2). An even higher percentage of human MRSA (88%) harbor one SCC*mec* belonging to type IV. Moreover, the SCC*mec* in seven of the eight bovine and human MRNAS also belong to types IV or V (Table 2). These results are similar to the published literature [22,23] with a high prevalence of type IV in CA-MRSA (like the human SA isolates of this study) and of both types IV and V in bovine LA-MRSA, with the other types present at lower prevalences. As far as MRSA from other sources are concerned, the SCC*mec* of three of the four Belgian isolates from boar, horses and fomites also belong to type IV and V (Table 2). Conversely, the SCC*mec* type II is predominant among Japanese isolates from dogs and cats (four out of five). As already published by others, the SCC*mec* type II is indeed the most prevalent in Japan [24]. It is also common in clinical samples from HA-MRSA and CA-MRSA in Asia and its presence in dogs and cats may be related to the proximity of these animal species with humans [25].

The only MRS in which neither mPCR nor WGS could identify the SCC*mec* type was one bovine *S. haemolyticus*. Indeed, genomic analysis did not confirm the presence of any SCC*mec* in this MRNAS, since no *ccr* complex could be identified and since SCC*mec* types are defined based on the *ccr* and *mec* complex elements [11,26]. Moreover, the detection of the IS*1272* in the genomic structure of this strain makes it different from the ancestral *mec* structure of *S. fleuretti*, *S. sciuri* or *S. vitulinus*. This strain is nevertheless of interest, not only in livestock where NAS are also responsible for subclinical bovine mastitis cases [27,28], but also potentially in public health in general, since mastitis-causing NAS could potentially also be responsible for different infections in other animal species and in humans [13,29]. 

Before the identification procedure of the types of SCC*mec*, MRS had to be identified and the different tests used showed some limits related to different factors. Of the 78 presumptive MRS(A) obtained after screening on Chrom MRSA ID^®^, only 68 MRS(A) were confirmed by disc diffusion assay and MIC test strips with cefoxitin (Table 1). As described by Van Griethuysen and collaborators, the *mecA* gene is lost in 14.4% of MRS(A) after 2 years of storage at −80 °C [30]. The isolates used in this study were screened on Chrom MRSA ID^®^ in 2014 and then confirmed with a cefoxitin susceptibility test in 2016 after storage at −80 °C. This may explain the loss of the cefoxitin resistance by these 10 presumptive MRS(A), if this resistance was due to the presence of a *mec* gene. Moreover, false positive or negative results can occur with the use of the cefoxitin disc diffusion assay for the detection of methicillin resistance [31]. The three SA isolates that were cefoxitin-resistant at either disc diffusion assay or MIC test strips, but *mecA*/*B*/*C* PCR negative could be explained by different hypotheses. At first, small interpretation errors of the results could have occurred since the results, at least for the two bovine SA are borderline (Table 1). Conversely, the disk diffusion assay of the human SA is not borderline and was similar after retesting. Secondly, staphylococci can use other resistance mechanisms, such as β-lactamase hyper-production, mutations at PBP-encoding or other genes (f.i., mutations at *gdpP* or *yjbH* genes induce PBP4 overproduction), the presence of other *mec* genes like *mecD* that was not looked for or still undescribed, or cell wall defective forms with intrinsic β-lactam resistance [32]. Further genomic analysis of these three isolates may bring answers to these questions. Meanwhile, the results suggest that the Chrom MRSA ID^®^ should be used as a first-line screening test, while the phenotypic resistance tests to cefoxitin and the *mec* gene PCR as second-line confirmatory tests. 

## 4. Conclusions

In conclusion, the results of this study confirm: (i) the presence of a majority of SCC*mec* types IV and V in bovine mastitis-associated MRSA and MRNAS, like in human CA-MRSA [22,23]; (ii) the strengths and limits of phenotypic and genetic tests for the identification of MRS and for SCC*mec* typing; and (iii) the need of more studies on MRNAS considering the potential of SCC*mec* interspecies transferability and the increasing impact of NAS in public health [13,29].

## 5. Materials and Methods

### 5.1. Isolate Collections and MRS Phenotypic Identification

A total of 1462 staphylococci isolated between 2005 and 2014 from human (n = 51), bovine milk (n = 1160), dog (n = 185), cat (n = 50), wild boar (n = 6), horse (n = 1), deer (n = 1), hare (n = 1), roe (n = 3) and fomites from an equine clinic (n = 4) were included in this study. Of these 1462 staphylococci, 723 originated from Belgium [23,33,34], 45 from Italy, 25 from Switzerland, 90 from Canada [35], 233 from Japan [36], 256 from Niger [37] and 90 from Senegal [38].

Although several of these staphylococci had already been studied for MR, all the 1462 isolates were screened for methicillin resistance with the Chrom MRSA ID^®^ agar (BioMérieux, Craponne, France). To confirm the resistance to methicillin, disc diffusion assays were performed on the growing isolates with the cefoxitin discs (Neo-sensitabs, Rosco Diagnostica, Taastrup, Denmark) (30 µg/mL) and the minimal inhibitory concentrations (MIC) were assessed by MIC test strips (Liofilchem Diagnostic, Roseto degli Abruzzi, Italy) (0.16–256 µg/mL) on Mueller–Hinton agar (Oxoid, Cambridge, UK) with a 0.5 McFarland bacterial suspension. After overnight incubation at 37 °C, inhibition zones were measured using the SIRSCAN micro device (i2a, Montpellier, France) for cefoxitin discs and manually for the MIC test strips, and interpreted according to the Clinical and Laboratory Standards Institute recommendations [39]: the inhibition zone for resistant SA is ≤21 mm and ≥22 mm for sensitive SA while the diameter is ≤24 mm for resistant NAS and ≥25 mm for sensitive NAS; the MIC for resistant SA is >4 µg/mL and ≤4 µg/mL for sensitive SA, while they are >0.25 µg/mL for resistant NAS and ≤0.25 µg/mL for sensitive NAS. The quality control strains where the ATCC 29,213 methicillin-sensitive *Staphylococcus aureus*, the ATCC 4330 MRSA for the *mecA* gene and the ATCCBAA-2312 MRSA for the *mecC* gene. 

### 5.2. MRS Species Identification

The species identity of the isolates growing on Chrom MRSA ID^®^ agar was confirmed with an Autoflex Biotyper Mass Spectrometer (MALDI-TOF MS^®^, Bruker Daltonics, Bremen, Germany) using the direct transfer method and α-cyano-4-hydroxycinnamic acid as matrix, according to the manufacturer’s instructions. In case of no peak detection, the samples were retested with the extended direct transfer method, using either on-target formic acid treatment or full ethanol-formic acid extraction. The spectra were analyzed with the MBT (MALDI BioTyper) Compass software version 4.1. (Bruker Daltonics, Bremen, Germany), which includes reference databases of 8252 bacterial and fungal species, expanded with 13 MSP covering 8 species of coagulase-negative staphylococci as previously described [40]. Only the identifications with a score value between 2.00 and 3.00 (green color) were considered.

### 5.3. PCR for *mec* Gene and SCC*mec* Identification

The DNA of the MRS(A) isolates were extracted following a protocol adapted from Unal and collaborators [41]. One colony was suspended into 50 µL of lysostaphin (0.1 mg/mL) (Sigma-Aldrich, Overijse, Belgium) and incubated at 37 °C for 10 min. Then, a mix of 45 µL of sterile water, 5 µL of proteinase K (2 mg/mL) (Sigma–Aldrich, Overijse, Belgium) and 150 µL of TRIS-HCL (0.1 M, pH 8) was added to the suspension and incubated at 56 °C for 10 min then at 95 °C for 5 min and centrifuged at 13,000× *g* for 5 min. Supernatants were recovered and stored at −20 °C until further use.

Two duplex PCR (*mecA/nuc* and *mecC/16S rDNA* staph) and one uniplex PCR (*mecB*) were performed with the multiplex PCR (mPCR) kit^®^ (QIAGEN, Venlo, The Netherlands) using 1.5 µL of DNA suspension. For each PCR, the MIX contained 7.5 µL of PCR reaction mix 2x, 3 µL of Q solution and 0.375 µL of each primer (10 µM) in a total volume of 13.5 µL. The targeted genes, primer sequences and PCR product lengths are reported in Table 3 [41,42,43]. The PCR amplification conditions were the same for the two duplex PCR: an initial denaturation step at 95 °C for 15 min; 35 cycles at 95 °C for 30 s (denaturation), 57 °C for 90 s (annealing) and 72 °C for 90 s (extension); and a final extension step at 72 °C for 10 min. Only the annealing temperature was different for the *mecB* PCR, at 59.7 °C instead of 57 °C. 

The SCC*mec* typing was based on a set of 5 multiplex PCR (mPCR) targeting the *ccr* and *mec* gene complexes and performed following an adapted protocol from Argudín and collaborators [42] using the multiplex PCR kit^®^ (QIAGEN, Venlo, The Netherlands). For each mPCR, 2 µL of DNA obtained by boiling was used and the mix was constituted of 7.5 µL of PCR reaction mix 2x, 3 µL of Q solution, 5µL of primer mix (10 µM). The targeted genes, primer sequences and the PCR product lengths (bp) are reported in the Table 4 [43,44,45,46]. The PCR amplification conditions for PCR1a and PCR1b were an initial denaturation at 95 °C for 15 min, 40 cycles of 95 °C for 30 s (denaturation), 57 °C for 90 s (annealing), and 72 °C for 2 min (extension), and a final extension at 72 °C for 10 min. For PCRs 2, 3 and 4 the conditions were different, with 35 cycles of 94 °C for 2 min (denaturation), 60 °C of 90 s (annealing) and 72 °C for 3 min (extension). All PCR amplified fragments were visualized by electrophoresis in 1.5% (*w*/*v*) agarose gel after staining with Midori green Advance DNA Stain (Nippon Genetics, Düren, Germany).

### 5.4. WGS 

Bacterial DNA was extracted from the MRS(A) whose SCC*mec* could not be identified by mPCR using an overnight bacterial culture pellet suspended in 495 µL of buffer (75 Mm NaCl, 25 Mm EDTA pH 8, 20 Mm Tris HCl pH 7). After addition of 50 µL of lysostaphin (50 µg mL^−1^) and 10 µL of RNase (10 µg mL^−1^), the suspension was incubated for 1 h at 37 °C. Then, 50 µL of 10% SDS and 5 µL of proteinase K (100 µg mL^−1^) were added and the suspension was incubated for 1 h at 55 °C. After adding 200 µL of NaCl 5 M, 700 µL of chloroform/isoamyalcohol (24/1) and the ethanol precipitation, the dried pellet containing DNA was suspended in 20 µL MiliQ water and stored at −20 °C until further use. 

The genomic DNA libraries of MRS(A) were prepared for Illumina sequencing according to the manufacturer’s instructions using the Nextera XT kit and sequenced by the NovaSeq 6000 Sequencing System (Illumina, San Diego, CA, USA). The raw read sequences were assembled into contigs with the pipeline shovill 1.0.4 including trimmomatic 0.38 for the cleaning and annotated using Prokka 1.13.3 [47]. Nanopore MinION long-read sequencing was performed using the Rapid Barcoding Sequencing kit (Oxford Nanopore) for library preparation. After guppy_gpu base calling, assembly of nanopore reads was done using Canu 1.8. The Illumina reads were cleaned with trimmomatic 0.36 (ILLUMINACLIP:illumina_oligos_and_revcomp:2:30:5:1:true LEADING:3 TRAILING:3 MAXINFO:40:0.2 MINLEN:36 options) and aligned with Canu assembly using BWA 0.7.15-r1140. Pilon 1.23 was run on this alignment for preliminary corrections. The final result was the corrected consensus provided by Pilon.

The SCC*mec* identification was performed using SCC*mec*Finder 1.2 available from the Center for Genomic Epidemiology (https://cge.cbs.dtu.dk/, accessed on 21 February 2020) with a %ID threshold of 90% and minimum length of 60%. An SCC*mec* Finder confirmation was performed for the not matching sequences with a %ID threshold and a minimum length of 80% and 40% respectively [21,26,44].

## Figures and Tables

**Table 1:** Cefoxitin susceptibility test vs. *mecA* PCR on the 78 staphylococci growing on Chrom MRSA ID^®^ agar.

Country	Host Species	*Staphylococcus* Species	Cefoxitin R ^1^		Cefoxitin S ^2^		Total
			*mecA+*	*mecA−*	*mecA+*	*mecA−*	
Belgium	Cow	*aureus*	26	2 ^3^		3	31
		*epidermidis*	1				1
		*haemolyticus*	1				1
	Boar	*aureus*	1				1
	Horse	*aureus*	1				1
	Human	*aureus*	25	1 ^4^			26
		*capitis*	1				1
		*epidermidis*	5				5
	Fomites	*aureus*	2				2
Canada	Cow	*aureus*				1	1
Japan	Dog	*aureus*	1				1
	Cat	*aureus*	4				4
Niger	Cow	*aureus*				3	3
Total			68	3	0	7	78

^1^ R = resistant, ^2^ S = sensitive, ^3^ one isolate was sensitive in the disk diffusion assay (diameter = 25 mm), but resistant in the MIC (Minimal Inhibitory Concentration) test strip (MIC = 6 μg/mL) and the second isolate was resistant in the disk diffusion assay (diameter = 18 mm), but sensitive in the MIC test (MIC = 4 μg/mL), ^4^ this isolate was resistant in the disk diffusion assay (diameter = 9 mm), but sensitive in the MIC test (MIC = 4 μg/mL).

**Table 2:** Identification by multiplex PCR (mPCR) and whole genome sequencing (WGS) of the staphylococcal chromosome cassette *mec* (SCC*mec*) present in the 68 *mecA* PCR-positive staphylococci.

Country	Host Species	*Staphylococcus* Species	SCC*mec* Type					Not Typeable	TOTAL *mecA*+ Isolates
			II	III	IV	V	VII		
Belgium	Cow	*aureus*	6		13 (2 ^1^)	4	3		26
		*epidermidis*				1			1
		*haemolyticus*						1 ^2^	1
	Boar	*aureus*		1					1
	Horse	*aureus*				1			1
	Human	*aureus*		1	22 (4)	2			25
		*capitis*			1				1
		*epidermidis*			2	3			5
	Fomites	*aureus*			2				2
Japan	Dog	*aureus*	1						1
	Cat	*aureus*	3 (1)		1 (1)				4
**Total**			10 (1)	2	41 (7)	11	3	1	68

^1^ Numbers of isolates whose SCC*mec* could not be identified by multiplex PCR, but well by WGS; ^2^ no SCC*mec* could be identified in this *S. haemolyticus* by either multiplex PCR or by WGS.

**Table 3:** Primers used for the *mecA/nuc*, *mecC/16S rDNA* staph and *mecB* gene detection by PCR.

Genes	Primers	Primer Sequence 5′-3′	PCR Product Length (bp ^1^)	References
*mecA*	mecA1	AAAATCGATGGTAAAGGTTGGC	533	[43]
	mecA2	AGTTCTGCAGTACCGGATTTGC		
*nuc*	nuc1	GCGATTGATGGTGATACGGTT	279	[43]
	nuc2	AGCCAAGCCTTGACGAACTAAAGC		
*mecC*	mecC454-F	GTCCCTAACAAAACACCCAAAGA	454	This study
	mecC454-R	GAAGATCTTTTCCGTTTTCAGC		[42]
*16S rDNA* staph	16S RNA1	GTTATTAGGGAAGAACATATGTG	750	[43]
	16S RNA2	CCACCTTCCTCCGGTTTGTCACC		
*mecB*	mecB-for	TTAACATATACACCCGCTTG	279	[9]
	mecB-rev	TAAAGTTCATTAGGCACCTCC		

^1^ bp = base pair.

**Table 4:** Primers used for the SCC*mec* typing by multiplex PCR.

mPCR ^1^	Genes	Primers	Primer Sequence 5′-3′ ^3^	PCR Product Lengths (bp ^4^)	References
PCR1a	Type 1 *ccr* ^2^	α1-dege	AACCTATATCATYAATCAGTRCGT	695	[44]
		βc	ATTGCCTTGATAATAGCCITCT		
	Type 2 *ccr*	α2	TAAAGGCATCAATGCACAAACACT	937	[44]
		βc	ATTGCCTTGATAATAGCCITCT		
	Type 3 *ccr*	α3	AGCTCAAAAGCAAGCAATAGAAT	1791	[44]
		βc	ATTGCCTTGATAATAGCCITCT		
	Type 7 *ccr*	α1-dege	AACCTATATCATYAATCAGTRCGT	417	[44]
		ccr7.4-rev	ACATGCGCTGTAGTGCAGGG		
PCR1b	*mecA*	mA1	TGCTATCCACCCTCAAACAGG	286	[44]
		mA2	AACGTTGTAACCACCCCAAGA		
	Type 4 *ccr*	α4.2	GTATCAATGCACCAGAACTT	1287	[44]
		β4.2 dege	TTGCGACTCTCTTGRCGTTT		
	Type 5 *ccr*	γR	CCTTTATAGACTGGATTATTCAAAATAT	518	[44]
		γF	CGTCTATTACAAGATGTTAAGGATAAT		
PCR2	class A *mec*	mI6	CATAACTTCCCATTCTGCAGATG	1963	[44]
		mA7	ATATACCAAACCCGACAACTACA		
	class B *mec*	IS7	ATGCTTAATGATAGCATCCGAATG	2827	[44]
		mA7	ATATACCAAACCCGACAACTACA		
	class C2 *mec*	IS2	TGAGGTTATTCAGATATTTCGATGT	804	[44]
		mA7	ATATACCAAACCCGACAACTACA		
PCR3	*mec*A	mecA 1	AAAATCGATGGTAAAGGTTGGC	533	[43]
		mecA 2	AGTTCTGCAGTACCGGATTTGC		
	class C1a/C1b *mec*	ISF4	CGGATTTTCGCCATGCCACGA	1232/286	[46]
		mA7	ATATACCAAACCCGACAACTACA		
PCR4	Type 8 *ccr*	ccr8.4-fw	CTCAAGCGATACGGTCACAA	1388	[42]
		ccr8.3-rev	TCAGGCCTTTACGACGTTTT		
	class E *mec*	mecC-fw	TTTTGCCTCGCTCTGATTTT	1083	[42]
		mecR1-rv	GCCAAAAGACCATTGGATTC		
	*mec* C	mecALGA251MultiFP	GAAAAAAAGGCTTAGAACGCCTC	138	[45]
		mecALGA251MultiRP	GAAGATCTTTTCCGTTTTCAGC		

^1^ mPCR = multiplex PCR, ^2^ *ccr* = cassette chromosome recombinase-encoding gene, ^3^ R = A or G; I = Inosine; Y = C or T, ^4^ bp = base pair.

## Data Availability

All genomic data related to this project, including raw reads, are available via the NCBI BioProject PRJNA607920.

## Supplementary Materials

The following are available online at https://www.mdpi.com/2079-6382/10/3/256/s1, Suppl. Table S1: The different SCC*mec* type profiles based on the multiplex PCR; Suppl. Table S2: The different SCC*mec* type profiles based on genomic analysis with SCC*mec*Finder 1.2; Suppl. Table S3: Identification with SCC*mec*Finder 1.2 after WGS of the SCC*mec* types in the nine staphylococci untypeable with the multiplex PCR.
