# Supplementary material for: Comparison of the Staphylococcal Chromosome Cassette (SCC) mec in Methicillin-Resistant Staphylococcus aureus (MRSA) and Non-aureus Staphylococci (MRNAS) from Animals and Humans
Source: Antibiotics (Basel). 2021 Mar 4;10(3):256. doi: 10.3390/antibiotics10030256 (PMC7998684; doi:10.3390/antibiotics10030256)
Supplement: Supplementary file 1 [file antibiotics-10-00256-s001.pdf]

Supplementary material

Supplementary table S1: The different SCC*mec* type profiles based on the multiplex PCR (mPCR)

SCC*mec* types	PCR1 (a, b) [1]							PCR2			PCR3		PCR4		
	mecA (286bp)	type 1 *ccr* [2]	type 2 *ccr*	type 3 *ccr*	type 4 *ccr*	type 5 *ccr*	type 7 *ccr*	class A *mec*	class B *mec*	class C2 *mec*	*mecA* (533pb)	class C1 *mec*	*mecC*	class E *mec*	type 8 *ccr*
**I**	+	+							+		+				
**II**	+		+					+			+				
**III**	+			+				+			+				
**III+SCChg**	+			+		+		+			+				
**IV**	+		+						+		+				
**V**	+					+				+	+				
**VI**	+				+				+		+				
**VII**	+					+					+	+			
**VIII**	+				+			+			+				
**IX**	+	+								+	+				
**X**	+						+				+	+			
**XI**													+	+	+
**II/IV**	+		+					+	+		+				
**VII/X**	+					+	+				+	+			

[1] + = PCR positive result, [2] *ccr* = cassette chromosome recombinase-encoding gene

Supplementary table S2: The different SCC*mec* type profiles based on genomic analysis with SCC*mec*Finder 1.2. [1]

**SCC*mec* types**	***ccr* complex genes [2]**												**_mec_ complex genes**								**Reference strains**
	ccrA	***ccrB***	***ccrA1***	***ccrB1***	***ccrA2***	***ccrB2***	***ccrA3***	***ccrB3***	***ccrA4***	***ccrB4***	***ccrC***	***ccrC2***	***mecA***	***mecC***	***mecR1***	**Δ*mecR1* [3]**	**Ψ*mecR1* [4]**	***mecI***	**IS*431* [5]**	**IS*1272***	
**I (1B)**			+	+									+			+			+	+	NCTC10442
**II (2A)**					+	+							+		+			+	+		N315
**III (3A)(Hg)**							+	+			(+)	(+)	+				+	+	+		85/2082
**IVa (2B)**					+	+							+			+			+		CA05
**V (5C2)**											+	+	+			+			+(2x)		WIS
**VI (4B)**									+	+			+			+			+		HDE288
**VII (5C1)**											+	+	+			+			+(2x)		JCSC6082)
**VIII (4A)**	+								+	+			+		+			+	+		C10682
**IX (1C2)**		+	+										+			+			+(2x)		JCSC6943
**X (7C1)**		+	+										+			+			+(2x)		JCSC6945
**XI (8^E)**			+					+						+	+			+			LGA251
**XII (9C2)**			+									+	+			+			+(3x)		BA01611
**XIII (9A)**												+	+		+			+			SS-99-44
**New ?**													+							+	C128

[1] **+ =** more than 95% sequence homology, [2] *ccr* = cassette chromosome recombinase gene, [3] Δ*mecR1* = *mecR1* gene with a deletion in the C-terminal inducer domain, [4] Ψ*mecR1* = gene coding for truncated signal transducer protein MecR1, [5] IS = insertion sequence

<u>Supplementary table S3:</u> Identification with SCC*mec*Finder 1.2 after whole genome sequencing (WGS) of the SCC*mec* types in the 9 staphylococci untypeable with the multiplex PCR (mPCR)

***Staphylococus* species**	**Host species**	***ccr* complex genes** [1]		***mec* complex genes**				**Other**	**SCC*mec* type**
		ccrA2	***ccrB2***	***mecA***	***mecR1***	**Δ*mecR1*** [3]	***mecI***	**IS*1272*** [4]	
**B231** (*S. aureus)*	cow	99.93 [2]	100	100		100		100	IVa(2B)
**B524** (*S. aureus)*	cow	100	100	100		100		99.95	IVa(2B)
**C128** (*S. haemolyticus)*	cow			100				99.75	None
**EQ02** (*S. aureus)*	human	100	100	100		100		100	IVa(2B)
**EQ06** (*S. aureus)*	human	100	100	99.95		100		99.94	IVa(2B)
**EQ13** (*S. aureus)*	human	99.93	100	100		100		100	IVa(2B)
**EQ17** (*S. aureus)*	human	100	100	100		100		100	IVa(2B)
**J215** (*S. aureus)*	cat	100	100	100	100		100		II(2A)
**J231** (*S. aureus)*	cat	100	100	100				99.62	IVa(2B)

[1] *ccr* = cassette chromosome recombinase gene, [2] % homology of the identified genes to genes present in the reference strains (see Suppl. Table 2), [3] Δ*mecR1* = *mecR1* gene with a deletion in the C-terminal inducer domain, [4] IS = insertion sequence
